# Is there a link between physical function testing and ventral hernia size?

**DOI:** 10.1007/s00464-025-12224-z

**Published:** 2025-09-29

**Authors:** William Head, Divyaam Satija, Kiana Shannon, Peter Edwards, Savannah Renshaw, Elanna Arhos, Lai Wei, Stephanie Di Stasi, Ajit Chaudhari, Benjamin Poulose

**Affiliations:** 1 Department of Surgery, Center for Abdominal Core Health, The Ohio State University Wexner Medical Center, Columbus, OH USA; 2 Department of Physical Therapy and Human Movement Sciences, Feinberg School of Medicine, Northwestern University, Evanston, IL USA; 3 Department of Biomedical Informatics, Center for Biostatistics, College of Medicine, The Ohio State University Wexner Medical Center, Columbus, OH USA; 4 Sports Medicine Research Institute, The Ohio State University Wexner Medical Center, Columbus, OH USA; 5 Division of Physical Therapy, School of Health and Rehabilitation Sciences, College of Medicine, The Ohio State University Wexner Medical Center, Columbus, OH USA

**Keywords:** Functional capacity, Functional testing, Hernia size, Physical therapy, Ventral hernia

## Abstract

**Background:**

Clinical assessments of physical function are widely used to evaluate candidacy and predict outcomes for various procedures. This study investigates the relationship between ventral hernia size and physical function tests.

**Methods:**

A cohort study was performed on prospectively collected data as part of a randomized control trial Pilot Trial of Abdominal Core Rehabilitation To Improve Outcomes After Ventral Hernia Repair (ABVENTURE-P). Patients scheduled for ventral hernia repair were enrolled between 4/2022 and 8/2024. Patients completed the Five Times Sit-to-Stand (5 × STS) and Timed Up & Go (TUG) physical functional tests pre-operatively. Hernia width was then measured at the time of hernia repair. Associations between hernia width and physical function tests were assessed using Spearman’s rank correlation tests, and associations between European Hernia Society (EHS) classifications and physical function tests were assessed using one-way ANOVA.

**Results:**

A total of 112 patients were evaluated with a mean hernia width of 7 cm (SD 7 cm), classified by EHS as 47% W1, 29% W2, and 24% W3. No significant association was found between 5 × STS scores and hernia size when analyzed as a continuous variable (Spearman’s rho = 0.13, *p* = 0.19) or across EHS classifications (*p* = 0.12). Similarly, TUG scores showed no significant correlation with hernia size as a continuous variable (Spearman’s rho = 0.13, *p* = 0.16) or across EHS classifications (*p* = 0.08).

**Conclusion:**

Physical function test scores alone did not correlate with ventral hernia size in this cohort. Hernia width was not significantly associated with 5 × STS or TUG performance when analyzed as a continuous variable by EHS classification.

A ventral hernia is a defect in the anterior abdominal wall through which intra-abdominal contents can protrude. These hernias are among the most common surgical conditions, with spontaneous defects affecting approximately 20% of adults and incisional defects occurring in up to 30% of patients following abdominal surgery [1]. In the United States, more than 600,000 ventral hernia repairs are performed annually, contributing to a significant economic burden, with repair costs alone estimated at ~ $9.7 billion per year [2]. Beyond financial implications, ventral hernias can profoundly impact patients’ quality of life, leading to chronic pain, poor cosmesis, and functional limitations [3, 4]. At this point in time, the relationship between ventral hernia size and functional limitations remains poorly understood.

Functional limitations refer to impairments in an individual's ability to perform daily activities due to an underlying health condition. Physical function tests, which assess mobility, strength, balance, and overall functional status, can be used to evaluate patients in the perioperative setting [5]. Specialties such as orthopedics and cardiothoracic surgery frequently employ these tests pre-operatively to assess surgical readiness and optimize patient care [6–11]. Additionally, in geriatric populations, these tests can not only predict post-operative outcomes but also inform interventions aimed at improving them [12–14]. While few studies have examined how hernias in general affect endurance or self-reported function, none have evaluated whether pre-operative physical function test scores can predict anatomical size of ventral hernias specifically.

In recent years, there has been growing recognition of the importance of abdominal core integrity in surgical decision-making and patient outcomes [15]. Understanding the relationship between ventral hernia size and physical function tests may enhance pre-operative risk stratification and patient counseling. If a correlation does exist, physical function tests could serve as a proxy for hernia size, aiding in surgical planning or identifying patients who benefit from prehabilitation. This study aims to evaluate this relationship, with the hypothesis that patients with poorer physical function test performance will have larger hernia defects.

## Materials and methods

The Biomedical Institutional Review Board of The Ohio State University approved this study (2021H0336).

### Study population

A prospective cohort study was performed with patients enrolled in the Pilot Trial of Abdominal Core Rehabilitation To Improve Outcomes After Ventral Hernia Repair (ABVENTURE-P) (NCT05142618) [16]. The ABVENTURE-P trial is a prospective randomized controlled trial evaluating the effect of a pre-operative core strengthening program on post-operative outcomes in patients undergoing ventral hernia repairs. These patients were evaluated for hernia repair through The Ohio State Wexner Medical Center’s Center for Abdominal Core Health. Inclusion criteria included age 18–70 years old, diagnosis of ventral hernia (primary or secondary), scheduled for elective ventral hernia repair, independent functional status, and transverse hernia width of ≥ 2 cm. Ventral hernia diagnosis was based on initial physician evaluation (e.g., through physical examination, CT scans, ultrasounds) and included the following defects: incisional, parastomal, epigastric, umbilical, and spigelian. Functional status was measured at the time of pre-operative study enrollment in the outpatient clinic setting no longer than 30 days prior to surgery [17]. Independent functional status was defined as a patient’s ability to require no help or support with activities of daily living and does not require/utilize a mobility assistance device within these 30 days. Exclusion criteria included previously diagnosed movement or balance disorder, use of ambulatory assistance device (i.e., walker or cane), and currently undergoing physical therapy or other skilled exercise intervention supervised by a medical rehabilitation professional at the time of pre-surgical functional measurements. Patients were enrolled between April 2022 and August 2024.

### Study design

Before undergoing surgical repair, patients completed a series of physical function tests in an outpatient clinic setting. This study focused on two primary assessments: the Five Times Sit-to-Stand (5 × STS) and Timed Up & Go (TUG) [18, 19].

The 5 × STS test begins and ends with the patient seated in a standardized, armless chair. The test starts when the patient rises to a full standing position and then sits back down five times as quickly as possible, without using their arms for support. Timing begins at the patient’s initial movement and stops when their buttocks makes contact with the chair after the fifth rise. This test was selected for the ventral hernia population because it requires core engagement, strength, and endurance through repeated bending at the waist. In individuals with abdominal core dysfunction, difficulty rising from a chair can significantly impact independent mobility and daily activities.

The TUG test also begins and ends with the patient seated in a chair. Upon command, the patient stands up, walks 3 m, turns 180˚, returns to the chair, and sits back down. Patients are instructed to complete the task as quickly and safely as possible. The test requires minimal equipment—a chair, a stopwatch, and a 4-m stretch of open space—making it simple to administer in a clinical setting. It was chosen for ventral hernia patients because it assesses multiple aspects of mobility, including bending, walking, and turning, which may be influenced both directly and indirectly by the hernia. In healthy, independent adults < 60 years old, a time of less than 10 s is generally considered normal for both 5 × STS and TUG [20–22]. Times ≥ 10 s for 5 × STS and ≥ 12 s for TUG are associated with increased risk of disability and fall, respectively.

These assessments were administered according to standard procedures; no study-specific a priori protocol was developed beyond the parent trial protocol. Following the completion of these functional tests, patients proceeded with surgical hernia repair. During the procedure, the largest transverse width of the hernia defect was measured in centimeters using a ruler. Transverse width was selected as the primary anatomical measurement for two primary reasons: (1) it is the most consistently reported measurement in the literature, (2) the European Hernia Society (EHS) classification scheme for incisional abdominal wall hernias utilizes three semi-quantitative divisions based on width, and (3) it is the most clinically relevant measure of risk stratification, surgical planning, and predictive post-operative complications [23–26].

### Data analysis

Statistical expertise was available to the authors. Ajit Chaudhari PhD and Divyaam Satija BS performed the analyses. Hernia width was evaluated as both a continuous and ordinal variable. Continuous measurements were recorded in centimeters, while ordinal data were categorized according to the European Hernia Society (EHS) classification for incisional abdominal wall hernias [23]. The EHS classification divides hernias into three groups based on width: W1 (< 4 cm), W2 (≥ 4–10 cm), and W3 (≥ 10 cm). Shapiro–Wilk tests of normality were performed for the continuous variables of interest, and based on non-normal distributions Spearman’s rank correlations were used to test the associations between width and the physical function measures. One-way ANOVA was used to test the fixed effect of EHS classification on the physical function measures. No corrections for multiple comparisons were used. Statistical tests were performed in JMP (version 17 Pro, JMP LLC, Cary, NC) and R (version 3.6.2, R Core Team, Vienna, Austria).

## Results

### Demographics

132 patients were prospectively evaluated with 112 included in the study. The remaining 20 patients were initially enrolled but did not proceed to surgery due to various patient-specific reasons. Table 1 summarizes the demographic data of the included cohort, both overall and stratified by EHS classification. The study population was predominantly male (61%) and White (84%), with a mean age of 50 years (SD 10 years). The average hernia width was 7 cm (SD 7 cm), ranging from a minimum of 2 cm to a maximum of 38 cm. Hernia distribution according to EHS classification was as follows: 47% W1, 29% W2, and 24% W3.

**Table 1:** Demographics arranged by European Hernia Society (EHS) classification

	Overall (*n* = 112)	EHS 1 (*n* = 53)	EHS 2 (*n* = 32)	EHS 3 (*n* = 27)	*p*-value
Age (years) (mean [SD])	50.25 (10.35)	47.98 (11.23)	51.78 (9.24)	52.89 (9.10)	*0.031
BMI (kg/m^2^) (mean [SD])	33.36 (5.94)	32.80 (6.08)	32.99 (6.02)	34.87 (5.51)	0.190
Male sex (%)	68 (60.71%)	37 (33.04%)	16 (14.29%)	15 (13.39%)	0.160
White race (%)	94 (83.93%)	44 (39.29%)	27 (24.11%)	23 (20.54%)	0.712
Patients with comorbidity (%)	46 (41.07%)	21 (18.75%)	14 (12.50%)	11 (9.82%)	0.975
Hernia width (cm) (mean [SD])	7.04 (6.94)	2.47 (0.58)	6.47 (1.93)	16.70 (7.78)	* < 0.001

*BMI* body mass index; *Kg/m*^*2*^ kilogram per meter squared; Comorbidities were based on the pre-operative clinic assessment note and/or the patient’s active problem list in the electronic health record; comorbidities include the following: history of abdominal aortic aneurysm, history of abdominal wall surgical site infection, ascites, chronic obstructive pulmonary disease, congestive heart failure, diabetes, dialysis-dependence, dyspnea, hepatic insufficiency or liver failure, hypertension, immunosuppression (history of transplant, recent steroid and/or chemotherapy administration, and other recent immunosuppressive medication[s]), low back pain, nicotine use (current or former), pelvic floor dysfunction, and other; *p-value < 0.05 statistically significant

### Five times sit-to-stand (5 × STS)

**Fig. 1:**
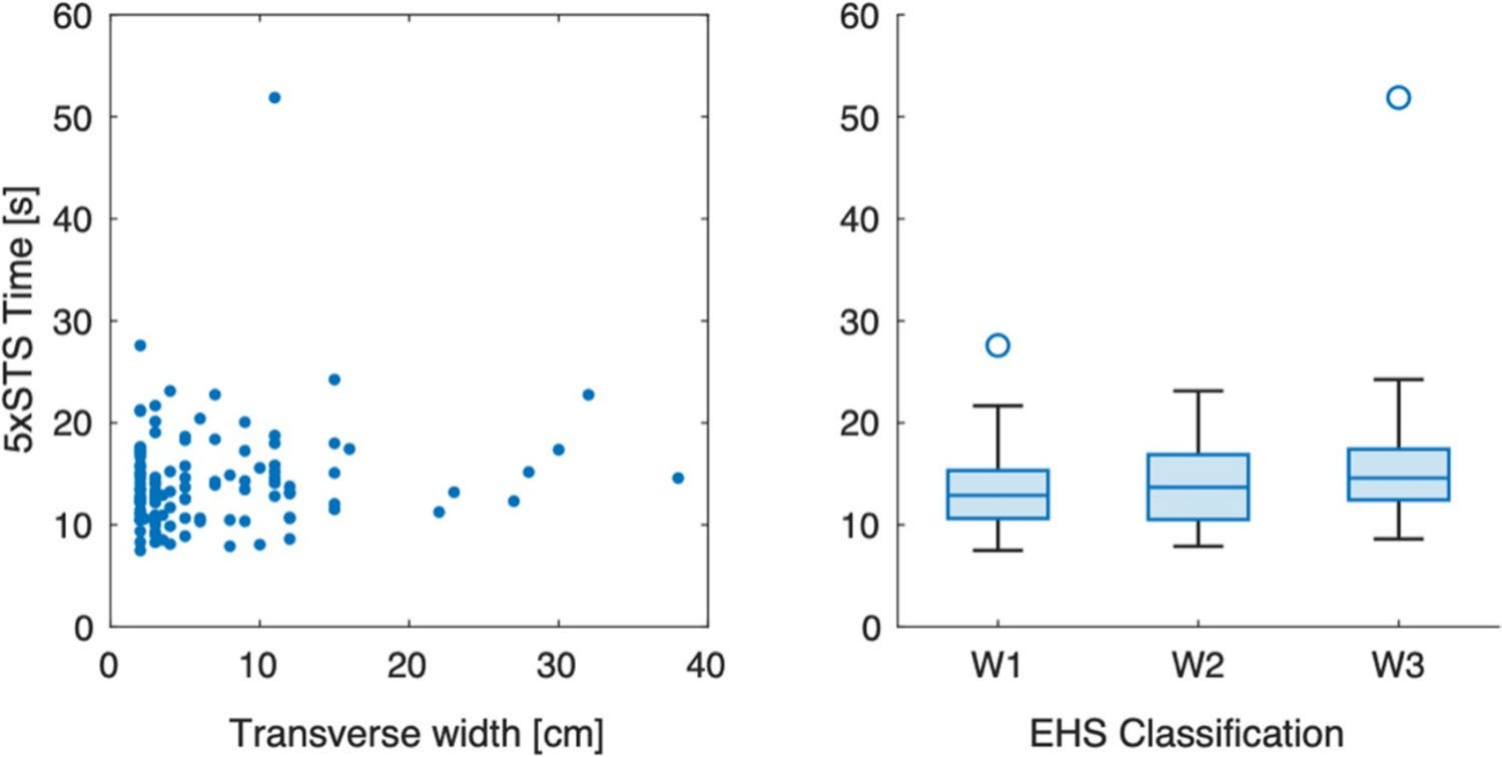
Scatter and box-and-whisker plot of five times sit-to-stand and hernia Width. *5 × STS* five times sit-to-stand; *EHS* European Hernia Society; W1 (< 4 cm), W2 (≥ 4–10 cm), and W3 (≥ 10 cm)

Figure 1 presents both a scatter plot (hernia width as a continuous variable) and box-and-whisker plot (EHS classification groups) for 5 × STS performance. No statistically significant correlation was observed between hernia width and 5 × STS (Spearman’s rho = 0.13, *p* = 0.19). Similarly, when analyzed by EHS classification, no significant difference was found (one-way ANOVA, *p* = 0.12).

### Timed Up & Go (TUG)

**Fig. 2:**
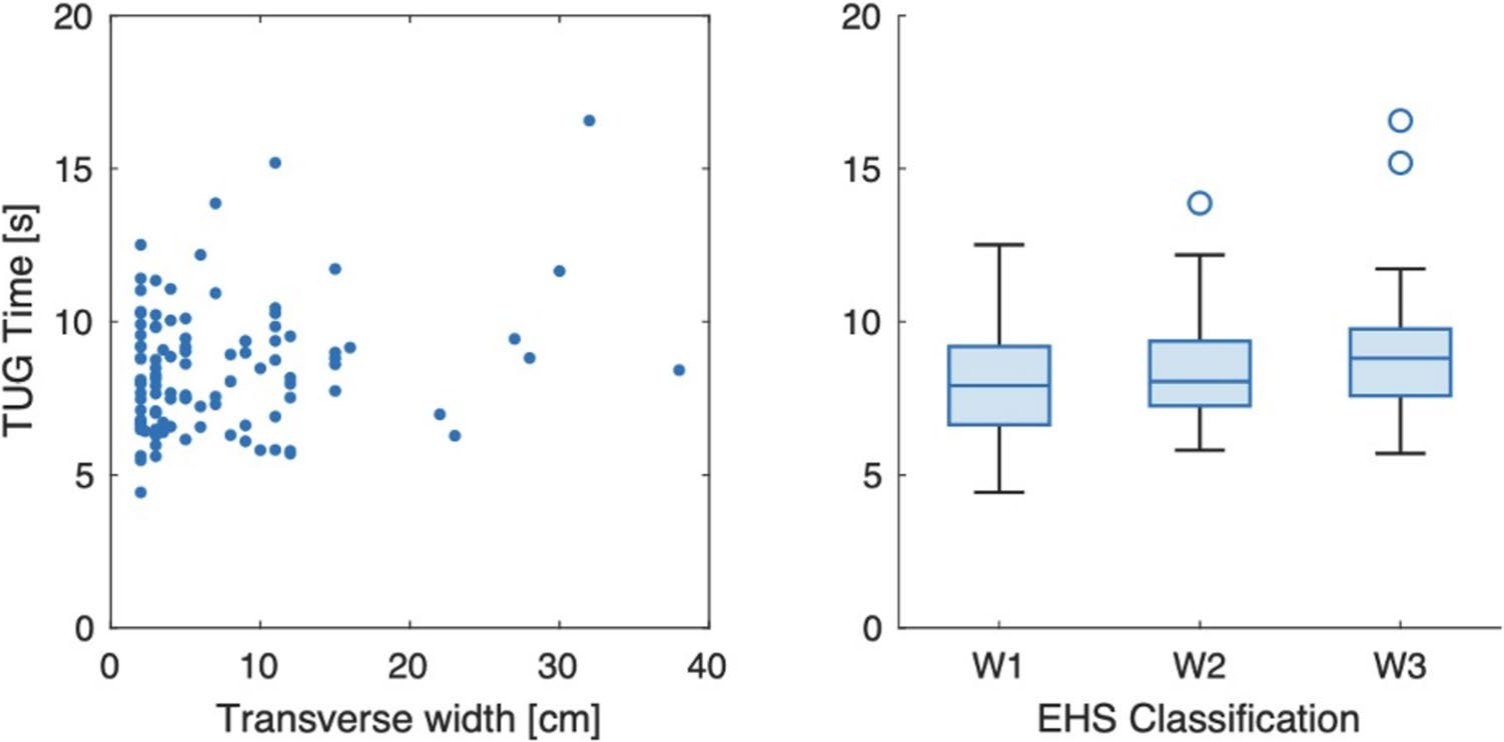
Scatter and box-and-whisker plot of timed up & go and hernia width. *TUG* timed up & go; *EHS* European Hernia Society; W1 (< 4 cm), W2 (≥ 4–10 cm), and W3 (≥ 10 cm)

Figure 2 provides scatter and box-and-whisker plots illustrating TUG performance based on hernia width and EHS classification. No significant correlation was identified between TUG and hernia width as a continuous variable (Spearman’s rho = 0.13, *p* = 0.16). Likewise, no significant difference was found among EHS classification groups (one-way ANOVA *p* = 0.08).

## Discussion

This study is the first to evaluate the relationship between physical function tests and ventral hernias. Specifically, it aimed to assess whether ventral hernia size, defined by transverse width, was associated with performance on two widely used physical function tests: Five Times Sit-to-Stand (5 × STS) and Timed Up & Go (TUG). Given the role of abdominal wall integrity in functional movement, it was hypothesized that poorer pre-operative test performance would correlate with larger hernia defects. However, the findings did not support this assumption. No statistically significant correlation was observed between hernia width—whether analyzed as a continuous variable or categorized by EHS classification—and functional test outcomes. These results ultimately suggest that hernia size alone is not a primary determinant of physical function in this population and that other factors may play a more significant role.

The use of physical function testing in the pre-operative setting is gaining attention as a means to assess disease severity and surgical risk. Various tests with objective outcomes have been studied across surgical specialties, with varying levels of supporting evidence. For example, Cardiopulmonary Exercise Testing (CPET) has been used to predict length of hospital stay, post-operative morbidity, and mortality not only in cardiothoracic surgery but also in patients undergoing intra-abdominal procedures [27–30]. Similarly, the Six-Minute Walk Test (6MWT) has been applied to assess functional capacity and predict surgical outcomes in a wide range of patient populations [31–33]. While these tests provide objective metrics, they may not fully capture the extent of a patient’s functional limitations, particularly in conditions affecting core integrity, such as ventral hernias.

Abdominal core integrity is a critical determinant of one’s physical function. Prior studies have demonstrated that the abdominal core is a central stabilizer of the trunk in static and dynamic activities, allowing for efficient force transfer, coordinated movement, and balance [34, 35]. Despite this central role, direct clinical evaluation of core function remains challenging due to its complex and integrated anatomical structure. In this study, we utilized the 5 × STS and TUG tests for two primary reasons: (1) their practicality and accessibility in clinical settings and (2) their involvement of core-related factors, such as trunk strength, endurance, and balance [18, 19]. Sensor-based monitoring of sit-to-stand performance has demonstrated associations between core movements and both objective and patient-reported functional status [36]. Similar sensor-based monitoring of TUG performance has linked phases such as sit-to-stand and turning with contractile properties of trunk and lower limb musculature [37]. As global assessments of physical function, 5 × STS and TUG were utilized in this study to capture the broad functional implications of impaired core integrity.

This study contributes to the growing literature evaluating the impact of hernias on functional outcomes. While prior research has evaluated other hernia categories and function impairment, this is the first study to evaluate objective physical function test outcomes across ventral hernia patients [38]. Our results showed a lack of association between hernia width and test performance, suggesting that anatomic size is not the primary driver of functional impairment in the ventral hernia population. Potential reasoning is that ventral hernia patients may develop compensatory mechanisms or that core dysfunction may exist independent of defect size. Another consideration is that our study sample did not have the appropriate power to generate a statistically significant conclusion, and thus, further study may be needed to evaluate these relationships better. Future studies should also explore more targeted measures of core function, such as The Quiet Unstable Sitting Test developed by our Center, and assess how these correlate with patient-reported outcomes, surgical risk, and recovery trajectories [39]. Another important area of consideration is patient-reported outcomes.

Subjective assessments of physical function have previously been evaluated, with some studies suggesting that patient-reported outcomes may provide a more accurate reflection of disease burden and functional impairment. Surveys such as the Short Form-36 Health Survey (SF-36), Patient-Reported Outcome Measures for Physical Function (PROM-PF), Clinical Frailty Scale (CFS), and MD Anderson Symptom Inventory (MDASI) are among the most commonly cited tools for evaluating self-perceived functional capacity, and they have demonstrated associations with post-operative outcomes [32, 40–44]. While the study presented here evaluates physical function testing in the ventral hernia population, it does so only with respect to objective measures. It is possible that incorporating a broader range of functional assessments—including subjective measures—could provide a more comprehensive understanding of how hernias impact daily activity and overall wellbeing. Future research should aim to compare the utility of various physical function tests, both objective and subjective, across different surgical populations. Identifying the most relevant and predictive assessments for specific patient groups will be essential for refining pre-operative risk stratification and optimizing surgical outcomes. Various studies and systematic reviews have demonstrated that pre-operative physical function impacts outcomes for patients with ventral hernias [45–49]. In the context of ventral hernias, integrating patient-reported measures with objective performance tests may provide a more nuanced assessment of functional limitations and their relationship to hernia size.

A key consideration is the impact of hernia location on abdominal integrity. While patients in this study had hernias ≥ 2 cm in width, ventral hernias vary in type and anatomic distribution, spanning umbilical, epigastric, spigelian, and incisional defects. The specific location of the hernia, rather than its size alone, may exert a greater influence on functional impairment. For instance, defects involving the rectus abdominis versus the obliques, or those affecting the linea alba versus the linea semilunaris, may have different consequences for core stability and movement. While previous studies have explored patient demographics, surgical timing, and repair techniques, none have systematically examined the anatomic location of hernia defects as a predictor of function.

Traditional surgical education views the anterior abdominal wall as a collection of discrete structural units that function independently. In contrast, physical therapy and kinesiology perspectives conceptualize the anterior abdominal wall as part of the broader, dynamic abdominal core, which also includes the spine, flanks, diaphragm, and pelvic floor. This system plays a critical role in movement, stability, and neuromuscular control. From this perspective, functional impairment is not solely dictated by the presence of a hernia but also by the coordination of core muscles and compensatory adaptations in surrounding muscle groups, such as the paraspinal, gluteal, and hip flexor muscles. Embracing the concept of the abdominal core as an interconnected system, rather than a set of isolated structures requiring repair, may offer hernia surgeons a more comprehensive approach. By adopting this interdisciplinary perspective, there is potential to enhance functional outcomes through pre- and post-operative rehabilitation while also tailoring surgical interventions to a patient’s specific functional needs for overall wellbeing. Specific optimization strategies may be informed by efforts including this study and randomized controlled trials such as ABVENTURE-P in addition to interdisciplinary discussion between surgeons and physical therapists.

Several limitations to this study should be noted. First, the inclusion and exclusion criteria were designed to enroll patients with relatively preserved function from a general health standpoint. Excluding patients over 70 years old, as well as those using an ambulatory assist device or with a history of prior physical therapy, may limit the generalizability of the findings to individuals with less functional impairment at baseline. Loss of domain was also not specifically evaluated as a separate hernia severity category. Second, the study was prospectively conducted at a single institution with a limited sample size, and the cohort was predominantly white and male, which may limit the generalizability of the findings. Expanding the patient pool and incorporating additional institutions with more diverse populations and expertise in ventral hernia management would likely enhance both the statistical power and external validity of the results. Third, functional test scores were only collected at a single pre-operative time point. Administering tests at multiple intervals, such as during a pre-operative clinic visit and on the day of surgery, would have allowed for score averaging and perhaps provided a more accurate representation of functional status. Fourth, only two physical function tests were used. Although these are among the most frequently utilized tests, there may be others that better reflect impairment due to ventral hernias. Finally, the study did not account for any temporal relevance or location of ventral hernias. It is possible that patients with chronic hernias may have developed compensatory mechanisms over time and therefore improved function compared to those with more acute defects. Additionally, the specific location of the hernia may also influence functional adaptations over time.

In conclusion, while this study did not demonstrate a significant relationship between physical function tests and ventral hernia size, it highlights the complexity of functional impairment in this patient population. The lack of correlation suggests that variability in pre-operative physical performance among ventral hernia patients is likely multifactorial and not solely driven by hernia size. Therefore, a more comprehensive approach that integrates physical function tests, patient-reported outcomes, and biomechanical analyses—alongside traditional imaging-based modalities—may be necessary. Future research should explore other relevant factors in the pre-operative setting and evaluate post-operative functional outcomes to enhance risk stratification and optimize surgical care for ventral hernia patients.
